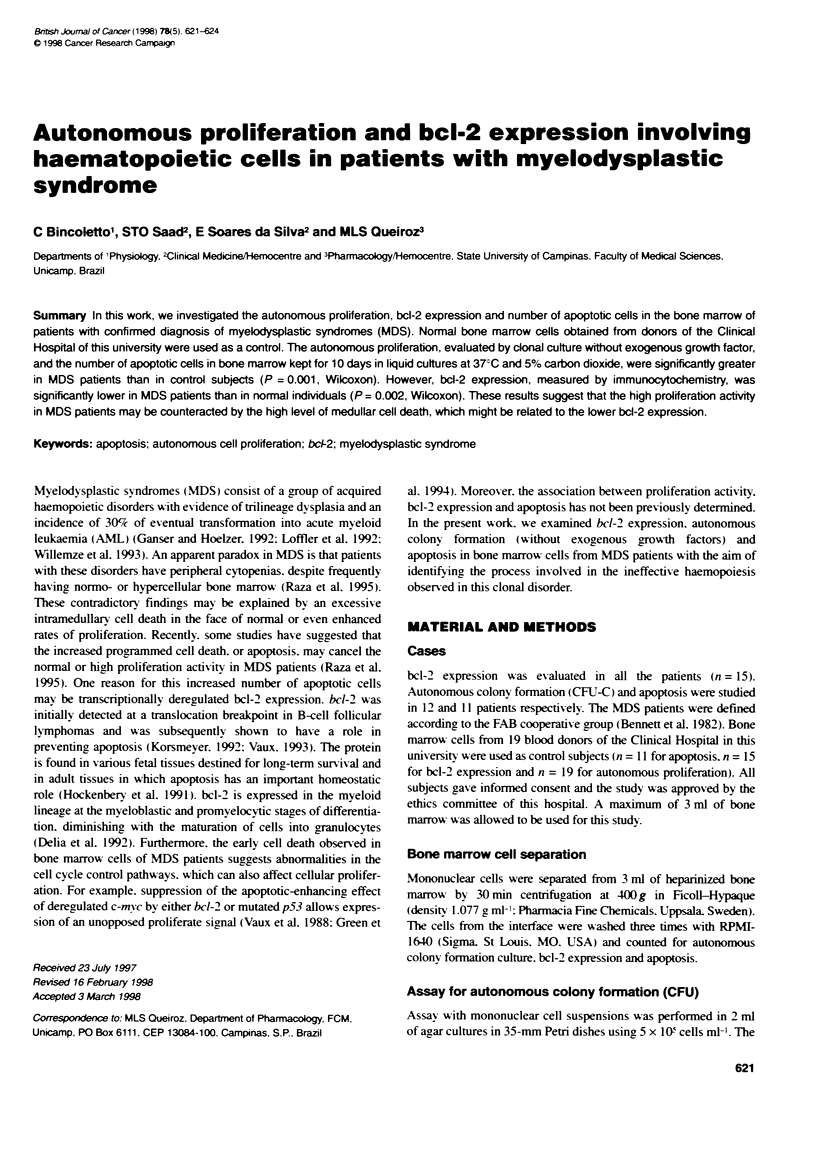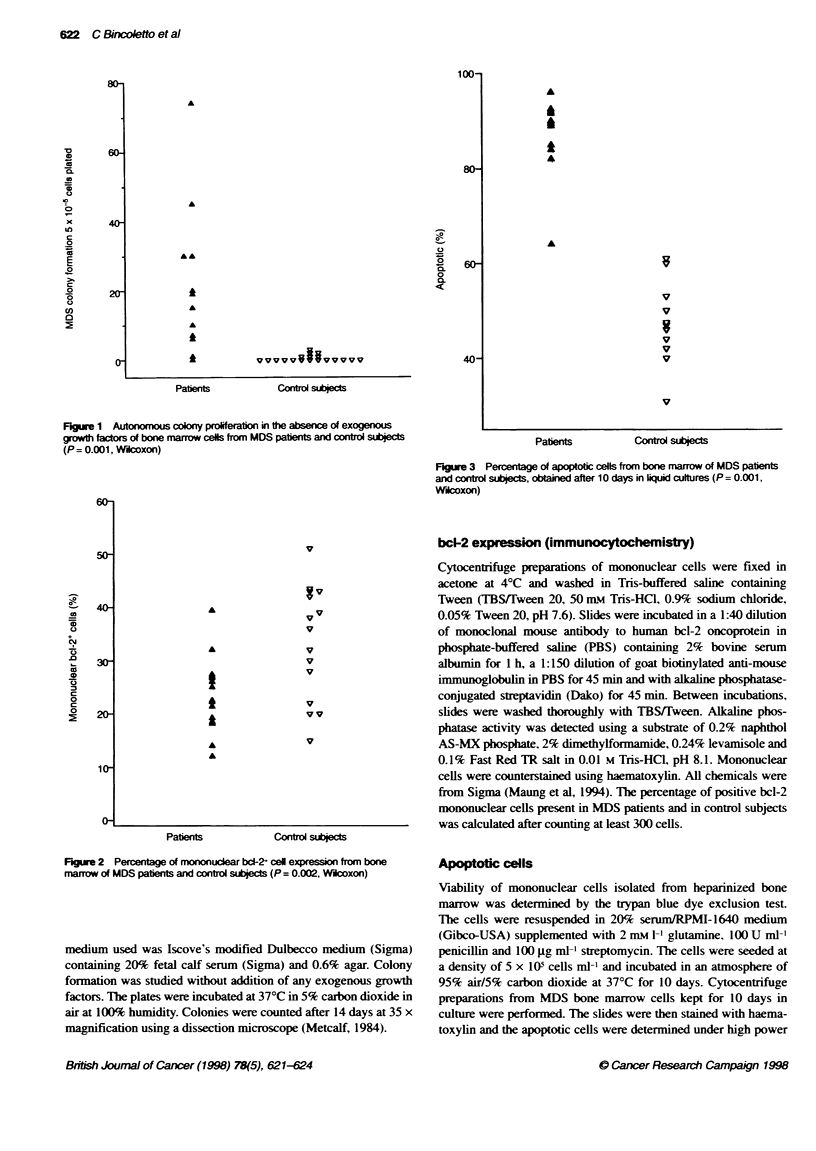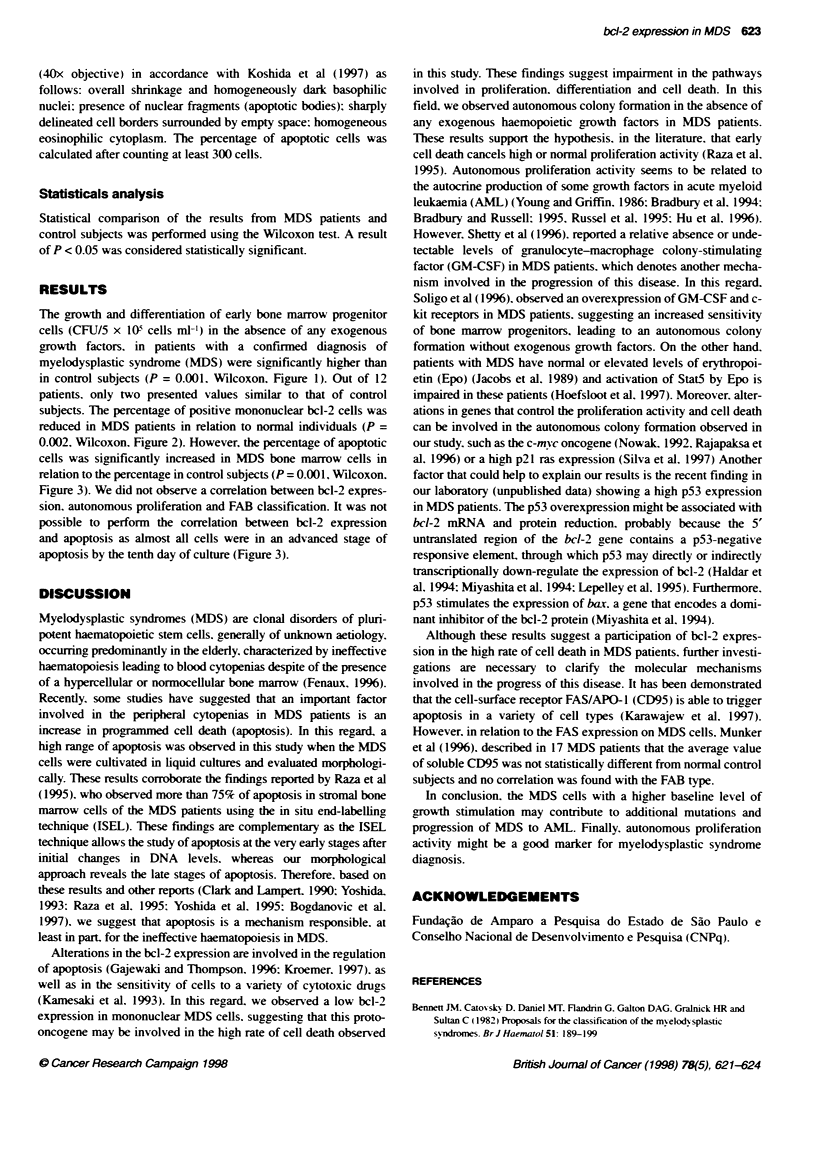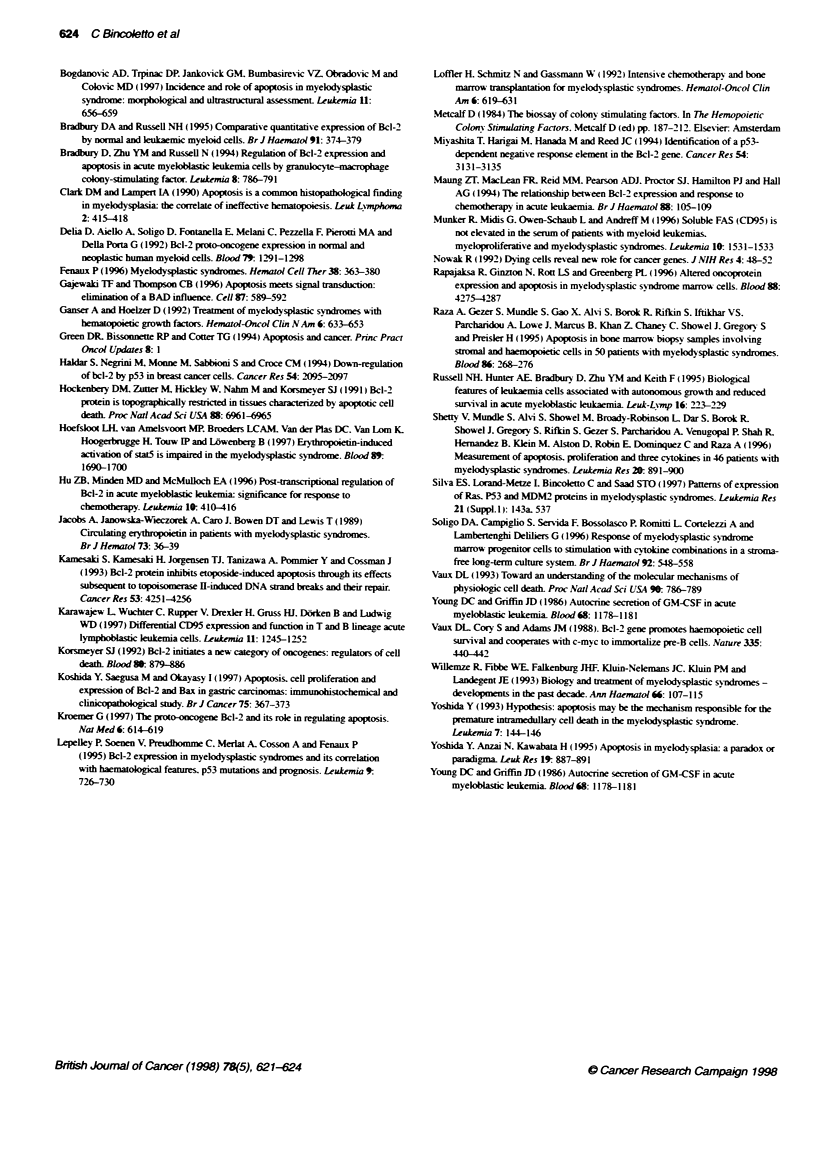# Autonomous proliferation and bcl-2 expression involving haematopoietic cells in patients with myelodysplastic syndrome.

**DOI:** 10.1038/bjc.1998.551

**Published:** 1998-09

**Authors:** C. Bincoletto, S. T. Saad, E. Soares da Silva, M. L. Queiroz

**Affiliations:** Department of Physiology, State University of Campinas, Faculty of Medical Sciences, Unicamp, Brazil.

## Abstract

In this work, we investigated the autonomous proliferation, bcl-2 expression and number of apoptotic cells in the bone marrow of patients with confirmed diagnosis of myelodysplastic syndromes (MDS). Normal bone marrow cells obtained from donors of the Clinical Hospital of this university were used as a control. The autonomous proliferation, evaluated by clonal culture without exogenous growth factor, and the number of apoptotic cells in bone marrow kept for 10 days in liquid cultures at 37 degrees C and 5% carbon dioxide, were significantly greater in MDS patients than in control subjects (P = 0.001, Wilcoxon). However, bcl-2 expression, measured by immunocytochemistry, was significantly lower in MDS patients than in normal individuals (P = 0.002, Wilcoxon). These results suggest that the high proliferation activity in MDS patients may be counteracted by the high level of medullar cell death, which might be related to the lower bcl-2 expression.


British Journal of Cancer (1998) 78(5), 621–624
© 1998 Cancer Research Campaign

### Autonomous proliferation and bcl-2 expression involving haematopoietic cells in patients with myelodysplastic syndrome

**C Bincoletto[1], STO Saad[2], E Soares da Silva[2] and MLS Queiroz[3]**

Departments of [1]Physiology, [2]Clinical Medicine/Hemocentre and [3]Pharmacology/Hemocentre, State University of Campinas, Faculty of Medical Sciences, Unicamp, Brazil

**Summary** In this work, we investigated the autonomous proliferation, bcl-2 expression and number of apoptotic cells in the bone marrow of patients with confirmed diagnosis of myelodysplastic syndromes (MDS). Normal bone marrow cells obtained from donors of the Clinical Hospital of this university were used as a control. The autonomous proliferation, evaluated by clonal culture without exogenous growth factor, and the number of apoptotic cells in bone marrow kept for 10 days in liquid cultures at 37°C and 5% carbon dioxide, were significantly greater in MDS patients than in control subjects ($P = 0.001$, Wilcoxon). However, bcl-2 expression, measured by immunocytochemistry, was significantly lower in MDS patients than in normal individuals ($P = 0.002$, Wilcoxon). These results suggest that the high proliferation activity in MDS patients may be counteracted by the high level of medullar cell death, which might be related to the lower bcl-2 expression.

**Keywords:** apoptosis; autonomous cell proliferation; *bcl-2*; myelodysplastic syndrome

Myelodysplastic syndromes (MDS) consist of a group of acquired haemopoietic disorders with evidence of trilineage dysplasia and an incidence of 30% of eventual transformation into acute myeloid leukaemia (AML) (Ganser and Hoelzer, 1992; Loffler et al, 1992; Willemze et al, 1993). An apparent paradox in MDS is that patients with these disorders have peripheral cytopenias, despite frequently having normo- or hypercellular bone marrow (Raza et al, 1995). These contradictory findings may be explained by an excessive intramedullary cell death in the face of normal or even enhanced rates of proliferation. Recently, some studies have suggested that the increased programmed cell death, or apoptosis, may cancel the normal or high proliferation activity in MDS patients (Raza et al, 1995). One reason for this increased number of apoptotic cells may be transcriptionally deregulated bcl-2 expression. *bcl-2* was initially detected at a translocation breakpoint in B-cell follicular lymphomas and was subsequently shown to have a role in preventing apoptosis (Korsmeyer, 1992; Vaux, 1993). The protein is found in various fetal tissues destined for long-term survival and in adult tissues in which apoptosis has an important homeostatic role (Hockenbery et al, 1991). bcl-2 is expressed in the myeloid lineage at the myeloblastic and promyelocytic stages of differentiation, diminishing with the maturation of cells into granulocytes (Delia et al, 1992). Furthermore, the early cell death observed in bone marrow cells of MDS patients suggests abnormalities in the cell cycle control pathways, which can also affect cellular proliferation. For example, suppression of the apoptotic-enhancing effect of deregulated c-*myc* by either *bcl-2* or mutated *p53* allows expression of an unopposed proliferate signal (Vaux et al, 1988; Green et al, 1994). Moreover, the association between proliferation activity, bcl-2 expression and apoptosis has not been previously determined. In the present work, we examined *bcl-2* expression, autonomous colony formation (without exogenous growth factors) and apoptosis in bone marrow cells from MDS patients with the aim of identifying the process involved in the ineffective haemopoiesis observed in this clonal disorder.

*Received 23 July 1997*
*Revised 16 February 1998*
*Accepted 3 March 1998*

*Correspondence to:* MLS Queiroz, Department of Pharmacology, FCM, Unicamp, PO Box 6111, CEP 13084-100, Campinas, S.P., Brazil

### MATERIAL AND METHODS

### Cases bcl-2 expression was evaluated in all the patients ($n = 15$). Autonomous colony formation (CFU-C) and apoptosis were studied in 12 and 11 patients respectively. The MDS patients were defined according to the FAB cooperative group (Bennett et al, 1982). Bone marrow cells from 19 blood donors of the Clinical Hospital in this university were used as control subjects ($n = 11$ for apoptosis, $n = 15$ for bcl-2 expression and $n = 19$ for autonomous proliferation). All subjects gave informed consent and the study was approved by the ethics committee of this hospital. A maximum of 3 ml of bone marrow was allowed to be used for this study.

### Bone marrow cell separation

Mononuclear cells were separated from 3 ml of heparinized bone marrow by 30 min centrifugation at 400 *g* in Ficoll–Hypaque (density 1.077 g ml$^{-1}$; Pharmacia Fine Chemicals, Uppsala, Sweden). The cells from the interface were washed three times with RPMI-1640 (Sigma, St Louis, MO, USA) and counted for autonomous colony formation culture, bcl-2 expression and apoptosis.

### Assay for autonomous colony formation (CFU)

Assay with mononuclear cell suspensions was performed in 2 ml of agar cultures in 35-mm Petri dishes using $5 \times 10^5$ cells ml$^{-1}$. The

**Figure 1** Autonomous colony proliferation in the absence of exogenous growth factors of bone marrow cells from MDS patients and control subjects ($P = 0.001$, Wilcoxon)

**Figure 2** Percentage of mononuclear bcl-2+ cell expression from bone marrow of MDS patients and control subjects ($P = 0.002$, Wilcoxon)

medium used was Iscove's modified Dulbecco medium (Sigma) containing 20% fetal calf serum (Sigma) and 0.6% agar. Colony formation was studied without addition of any exogenous growth factors. The plates were incubated at 37°C in 5% carbon dioxide in air at 100% humidity. Colonies were counted after 14 days at 35 × magnification using a dissection microscope (Metcalf, 1984).

**Figure 3** Percentage of apoptotic cells from bone marrow of MDS patients and control subjects, obtained after 10 days in liquid cultures ($P = 0.001$, Wilcoxon)

### bcl-2 expression (immunocytochemistry)

Cytocentrifuge preparations of mononuclear cells were fixed in acetone at 4°C and washed in Tris-buffered saline containing Tween (TBS/Tween 20, 50 mM Tris-HCl, 0.9% sodium chloride, 0.05% Tween 20, pH 7.6). Slides were incubated in a 1:40 dilution of monoclonal mouse antibody to human bcl-2 oncoprotein in phosphate-buffered saline (PBS) containing 2% bovine serum albumin for 1 h, a 1:150 dilution of goat biotinylated anti-mouse immunoglobulin in PBS for 45 min and with alkaline phosphatase-conjugated streptavidin (Dako) for 45 min. Between incubations, slides were washed thoroughly with TBS/Tween. Alkaline phosphatase activity was detected using a substrate of 0.2% naphthol AS-MX phosphate, 2% dimethylformamide, 0.24% levamisole and 0.1% Fast Red TR salt in 0.01 M Tris-HCl, pH 8.1. Mononuclear cells were counterstained using haematoxylin. All chemicals were from Sigma (Maung et al, 1994). The percentage of positive bcl-2 mononuclear cells present in MDS patients and in control subjects was calculated after counting at least 300 cells.

### Apoptotic cells

Viability of mononuclear cells isolated from heparinized bone marrow was determined by the trypan blue dye exclusion test. The cells were resuspended in 20% serum/RPMI-1640 medium (Gibco-USA) supplemented with 2 mM $l^{-1}$ glutamine, 100 U $ml^{-1}$ penicillin and 100 μg $ml^{-1}$ streptomycin. The cells were seeded at a density of $5 \times 10^5$ cells $ml^{-1}$ and incubated in an atmosphere of 95% air/5% carbon dioxide at 37°C for 10 days. Cytocentrifuge preparations from MDS bone marrow cells kept for 10 days in culture were performed. The slides were then stained with haematoxylin and the apoptotic cells were determined under high power

© Cancer Research Campaign 1998

(40× objective) in accordance with Koshida et al (1997) as follows: overall shrinkage and homogeneously dark basophilic nuclei; presence of nuclear fragments (apoptotic bodies); sharply delineated cell borders surrounded by empty space; homogeneous eosinophilic cytoplasm. The percentage of apoptotic cells was calculated after counting at least 300 cells.

### Statisticals analysis

Statistical comparison of the results from MDS patients and control subjects was performed using the Wilcoxon test. A result of $P < 0.05$ was considered statistically significant.

### RESULTS

The growth and differentiation of early bone marrow progenitor cells (CFU/5 × $10^5$ cells $ml^{-1}$) in the absence of any exogenous growth factors, in patients with a confirmed diagnosis of myelodysplastic syndrome (MDS) were significantly higher than in control subjects ($P = 0.001$, Wilcoxon, Figure 1). Out of 12 patients, only two presented values similar to that of control subjects. The percentage of positive mononuclear bcl-2 cells was reduced in MDS patients in relation to normal individuals ($P = 0.002$, Wilcoxon, Figure 2). However, the percentage of apoptotic cells was significantly increased in MDS bone marrow cells in relation to the percentage in control subjects ($P = 0.001$, Wilcoxon, Figure 3). We did not observe a correlation between bcl-2 expression, autonomous proliferation and FAB classification. It was not possible to perform the correlation between bcl-2 expression and apoptosis as almost all cells were in an advanced stage of apoptosis by the tenth day of culture (Figure 3).

### DISCUSSION

Myelodysplastic syndromes (MDS) are clonal disorders of pluripotent haematopoietic stem cells, generally of unknown aetiology, occurring predominantly in the elderly, characterized by ineffective haematopoiesis leading to blood cytopenias despite of the presence of a hypercellular or normocellular bone marrow (Fenaux, 1996). Recently, some studies have suggested that an important factor involved in the peripheral cytopenias in MDS patients is an increase in programmed cell death (apoptosis). In this regard, a high range of apoptosis was observed in this study when the MDS cells were cultivated in liquid cultures and evaluated morphologically. These results corroborate the findings reported by Raza et al (1995), who observed more than 75% of apoptosis in stromal bone marrow cells of the MDS patients using the in situ end-labelling technique (ISEL). These findings are complementary as the ISEL technique allows the study of apoptosis at the very early stages after initial changes in DNA levels, whereas our morphological approach reveals the late stages of apoptosis. Therefore, based on these results and other reports (Clark and Lampert, 1990; Yoshida, 1993; Raza et al, 1995; Yoshida et al, 1995; Bogdanovic et al, 1997), we suggest that apoptosis is a mechanism responsible, at least in part, for the ineffective haematopoiesis in MDS.

Alterations in the bcl-2 expression are involved in the regulation of apoptosis (Gajewaki and Thompson, 1996; Kroemer, 1997), as well as in the sensitivity of cells to a variety of cytotoxic drugs (Kamesaki et al, 1993). In this regard, we observed a low bcl-2 expression in mononuclear MDS cells, suggesting that this proto-oncogene may be involved in the high rate of cell death observed in this study. These findings suggest impairment in the pathways involved in proliferation, differentiation and cell death. In this field, we observed autonomous colony formation in the absence of any exogenous haemopoietic growth factors in MDS patients. These results support the hypothesis, in the literature, that early cell death cancels high or normal proliferation activity (Raza et al, 1995). Autonomous proliferation activity seems to be related to the autocrine production of some growth factors in acute myeloid leukaemia (AML) (Young and Griffin, 1986; Bradbury et al, 1994; Bradbury and Russell; 1995, Russel et al, 1995; Hu et al, 1996). However, Shetty et al (1996), reported a relative absence or undetectable levels of granulocyte–macrophage colony-stimulating factor (GM-CSF) in MDS patients, which denotes another mechanism involved in the progression of this disease. In this regard, Soligo et al (1996), observed an overexpression of GM-CSF and c-kit receptors in MDS patients, suggesting an increased sensitivity of bone marrow progenitors, leading to an autonomous colony formation without exogenous growth factors. On the other hand, patients with MDS have normal or elevated levels of erythropoietin (Epo) (Jacobs et al, 1989) and activation of Stat5 by Epo is impaired in these patients (Hoefsloot et al, 1997). Moreover, alterations in genes that control the proliferation activity and cell death can be involved in the autonomous colony formation observed in our study, such as the c-*myc* oncogene (Nowak, 1992, Rajapaksa et al, 1996) or a high p21 ras expression (Silva et al, 1997) Another factor that could help to explain our results is the recent finding in our laboratory (unpublished data) showing a high p53 expression in MDS patients. The p53 overexpression might be associated with *bcl*-2 mRNA and protein reduction, probably because the 5′ untranslated region of the *bcl*-2 gene contains a p53-negative responsive element, through which p53 may directly or indirectly transcriptionally down-regulate the expression of bcl-2 (Haldar et al, 1994; Miyashita et al, 1994; Lepelley et al, 1995). Furthermore, p53 stimulates the expression of *bax*, a gene that encodes a dominant inhibitor of the bcl-2 protein (Miyashita et al, 1994).

Although these results suggest a participation of bcl-2 expression in the high rate of cell death in MDS patients, further investigations are necessary to clarify the molecular mechanisms involved in the progress of this disease. It has been demonstrated that the cell-surface receptor FAS/APO-1 (CD95) is able to trigger apoptosis in a variety of cell types (Karawajew et al, 1997). However, in relation to the FAS expression on MDS cells, Munker et al (1996), described in 17 MDS patients that the average value of soluble CD95 was not statistically different from normal control subjects and no correlation was found with the FAB type.

In conclusion, the MDS cells with a higher baseline level of growth stimulation may contribute to additional mutations and progression of MDS to AML. Finally, autonomous proliferation activity might be a good marker for myelodysplastic syndrome diagnosis.

### ACKNOWLEDGEMENTS

Fundação de Amparo a Pesquisa do Estado de São Paulo e Conselho Nacional de Desenvolvimento e Pesquisa (CNPq).

#### REFERENCES

Bennett JM, Catovsky D, Daniel MT, Flandrin G, Galton DAG, Gralnick HR and Sultan C (1982) Proposals for the classification of the myelodysplastic syndromes. *Br J Haematol* 51: 189–199

© Cancer Research Campaign 1998

Bogdanovic AD, Trpinac DP, Jankovick GM, Bumbasirevic VZ, Obradovic M and Colovic MD (1997) Incidence and role of apoptosis in myelodysplastic syndrome: morphological and ultrastructural assessment. *Leukemia* **11**: 656–659

Bradbury DA and Russell NH (1995) Comparative quantitative expression of Bcl-2 by normal and leukaemic myeloid cells. *Br J Haematol* **91**: 374–379

Bradbury D, Zhu YM and Russell N (1994) Regulation of Bcl-2 expression and apoptosis in acute myeloblastic leukemia cells by granulocyte–macrophage colony-stimulating factor. *Leukemia* **8**: 786–791

Clark DM and Lampert IA (1990) Apoptosis is a common histopathological finding in myelodysplasia: the correlate of ineffective hematopoiesis. *Leuk Lymphoma* **2**: 415–418

Delia D, Aiello A, Soligo D, Fontanella E, Melani C, Pezzella F, Pierotti MA and Della Porta G (1992) Bcl-2 proto-oncogene expression in normal and neoplastic human myeloid cells. *Blood* **79**: 1291–1298

Fenaux P (1996) Myelodysplastic syndromes. *Hematol Cell Ther* **38**: 363–380

Gajewaki TF and Thompson CB (1996) Apoptosis meets signal transduction: elimination of a BAD influence. *Cell* **87**: 589–592

Ganser A and Hoelzer D (1992) Treatment of myelodysplastic syndromes with hematopoietic growth factors. *Hematol-Oncol Clin N Am* **6**: 633–653

Green DR, Bissonnette RP and Cotter TG (1994) Apoptosis and cancer. *Princ Pract Oncol Updates* **8**: 1

Haldar S, Negrini M, Monne M, Sabbioni S and Croce CM (1994) Down-regulation of bcl-2 by p53 in breast cancer cells. *Cancer Res* **54**: 2095–2097

Hockenbery DM, Zutter M, Hickley W, Nahm M and Korsmeyer SJ (1991) Bcl-2 protein is topographically restricted in tissues characterized by apoptotic cell death. *Proc Natl Acad Sci USA* **88**: 6961–6965

Hoefsloot LH, van Amelsvoort MP, Broeders LCAM, Van der Plas DC, Van Lom K, Hoogerbrugge H, Touw IP and Löwenberg B (1997) Erythropoietin-induced activation of stat5 is impaired in the myelodysplastic syndrome. *Blood* **89**: 1690–1700

Hu ZB, Minden MD and McMulloch EA (1996) Post-transcriptional regulation of Bcl-2 in acute myeloblastic leukemia: significance for response to chemotherapy. *Leukemia* **10**: 410–416

Jacobs A, Janowska-Wieczorek A, Caro J, Bowen DT and Lewis T (1989) Circulating erythropoietin in patients with myelodysplastic syndromes. *Br J Hematol* **73**: 36–39

Kamesaki S, Kamesaki H, Jorgensen TJ, Tanizawa A, Pommier Y and Cossman J (1993) Bcl-2 protein inhibits etoposide-induced apoptosis through its effects subsequent to topoisomerase II-induced DNA strand breaks and their repair. *Cancer Res* **53**: 4251–4256

Karawajew L, Wuchter C, Rupper V, Drexler H, Gruss HJ, Dörken B and Ludwig WD (1997) Differential CD95 expression and function in T and B lineage acute lymphoblastic leukemia cells. *Leukemia* **11**: 1245–1252

Korsmeyer SJ (1992) Bcl-2 initiates a new category of oncogenes: regulators of cell death. *Blood* **80**: 879–886

Koshida Y, Saegusa M and Okayasy I (1997) Apoptosis, cell proliferation and expression of Bcl-2 and Bax in gastric carcinomas: immunohistochemical and clinicopathological study. *Br J Cancer* **75**: 367–373

Kroemer G (1997) The proto-oncogene Bcl-2 and its role in regulating apoptosis. *Nat Med* **6**: 614–619

Lepelley P, Soenen V, Preudhomme C, Merlat A, Cosson A and Fenaux P (1995) Bcl-2 expression in myelodysplastic syndromes and its correlation with haematological features, p53 mutations and prognosis. *Leukemia* **9**: 726–730

Loffler H, Schmitz N and Gassmann W (1992) Intensive chemotherapy and bone marrow transplantation for myelodysplastic syndromes. *Hematol-Oncol Clin Am* **6**: 619–631

Metcalf D (1984) The biossay of colony stimulating factors. In *The Hemopoietic Colony Stimulating Factors*, Metcalf D (ed) pp. 187–212. Elsevier: Amsterdam

Miyashita T, Harigai M, Hanada M and Reed JC (1994) Identification of a p53-dependent negative response element in the Bcl-2 gene. *Cancer Res* **54**: 3131–3135

Maung ZT, MacLean FR, Reid MM, Pearson ADJ, Proctor SJ, Hamilton PJ and Hall AG (1994) The relationship between Bcl-2 expression and response to chemotherapy in acute leukaemia. *Br J Haematol* **88**: 105–109

Munker R, Midis G, Owen-Schaub L and Andreff M (1996) Soluble FAS (CD95) is not elevated in the serum of patients with myeloid leukemias, myeloproliferative and myelodysplastic syndromes. *Leukemia* **10**: 1531–1533

Nowak R (1992) Dying cells reveal new role for cancer genes. *J NIH Res* **4**: 48–52

Rapajaksa R, Ginzton N, Rott LS and Greenberg PL (1996) Altered oncoprotein expression and apoptosis in myelodysplastic syndrome marrow cells. *Blood* **88**: 4275–4287

Raza A, Gezer S, Mundle S, Gao X, Alvi S, Borok R, Rifkin S, Iftikhar VS, Parcharidou A, Lowe J, Marcus B, Khan Z, Chaney C, Showel J, Gregory S and Preisler H (1995) Apoptosis in bone marrow biopsy samples involving stromal and haemopoietic cells in 50 patients with myelodysplastic syndromes. *Blood* **86**: 268–276

Russell NH, Hunter AE, Bradbury D, Zhu YM and Keith F (1995) Biological features of leukaemia cells associated with autonomous growth and reduced survival in acute myeloblastic leukaemia. *Leuk-Lymp* **16**: 223–229

Shetty V, Mundle S, Alvi S, Showel M, Broady-Robinson L, Dar S, Borok R, Showel J, Gregory S, Rifkin S, Gezer S, Parcharidou A, Venugopal P, Shah R, Hernandez B, Klein M, Alston D, Robin E, Dominquez C and Raza A (1996) Measurement of apoptosis, proliferation and three cytokines in 46 patients with myelodysplastic syndromes. *Leukemia Res* **20**: 891–900

Silva ES, Lorand-Metze I, Bincoletto C and Saad STO (1997) Patterns of expression of Ras, P53 and MDM2 proteins in myelodysplastic syndromes. *Leukemia Res* **21** (Suppl.1): 143a, 537

Soligo DA, Campiglio S, Servida F, Bossolasco P, Romitti L, Cortelezzi A and Lambertenghi Deliliers G (1996) Response of myelodysplastic syndrome marrow progenitor cells to stimulation with cytokine combinations in a stroma-free long-term culture system. *Br J Haematol* **92**: 548–558

Vaux DL (1993) Toward an understanding of the molecular mechanisms of physiologic cell death. *Proc Natl Acad Sci USA* **90**: 786–789

Young DC and Griffin JD (1986) Autocrine secretion of GM-CSF in acute myeloblastic leukemia. *Blood* **68**: 1178–1181

Vaux DL, Cory S and Adams JM (1988). Bcl-2 gene promotes haemopoietic cell survival and cooperates with c-myc to immortalize pre-B cells. *Nature* **335**: 440–442

Willemze R, Fibbe WE, Falkenburg JHF, Kluin-Nelemans JC, Kluin PM and Landegent JE (1993) Biology and treatment of myelodysplastic syndromes – developments in the past decade. *Ann Haematol* **66**: 107–115

Yoshida Y (1993) Hypothesis: apoptosis may be the mechanism responsible for the premature intramedullary cell death in the myelodysplastic syndrome. *Leukemia* **7**: 144–146

Yoshida Y, Anzai N, Kawabata H (1995) Apoptosis in myelodysplasia: a paradox or paradigma. *Leuk Res* **19**: 887–891

Young DC and Griffin JD (1986) Autocrine secretion of GM-CSF in acute myeloblastic leukemia. *Blood* **68**: 1178–1181

© Cancer Research Campaign 1998